# Reducing symptoms of major depressive disorder through a systematic training of general emotion regulation skills: protocol of a randomized controlled trial

**DOI:** 10.1186/1471-244X-14-20

**Published:** 2014-01-27

**Authors:** Anna M Ehret, Judith Kowalsky, Winfried Rief, Wolfgang Hiller, Matthias Berking

**Affiliations:** 1 Department of Clinical Psychology and Psychotherapy, University of Marburg, Gutenbergstrasse 18, 35032 Marburg, Germany; 2 Department of Clinical Psychology and Psychotherapy, University of Mainz, Wallstraße 3, 55122 Mainz, Germany; 3 Division Health Trainings Online, Leuphana University Lueneburg, Innovation Incubator, Rotenbleicher Weg 67, 21335 Lueneburg, Germany

**Keywords:** Emotion regulation, Major depressive disorder, Treatment, Skills training, Randomized controlled trial

## Abstract

**Background:**

Major Depressive Disorder is one of the most challenging mental health problems of our time. Although effective psychotherapeutic treatments are available, many patients fail to demonstrate clinically significant improvements. Difficulties in emotion regulation have been identified as putative risk and maintaining factors for Major Depressive Disorder. Systematically enhancing adaptive emotion regulation skills should thus help reduce depressive symptom severity. However, at this point, no study has systematically evaluated effects of increasing adaptive emotion regulation skills application on symptoms of Major Depressive Disorder. In the intended study, we aim to evaluate stand-alone effects of a group-based training explicitly and exclusively targeting general emotion regulation skills on depressive symptom severity and assess whether this training augments the outcome of subsequent individual cognitive behavioral therapy for depression.

**Methods/Design:**

In the evaluation of the Affect Regulation Training, we will conduct a prospective randomized-controlled trial. Effects of the Affect Regulation Training on depressive symptom severity and outcomes of subsequent individual therapy for depression will be compared with an active, common factor based treatment and a waitlist control condition. The study sample will include 120 outpatients meeting criteria for Major Depressive Disorder. Depressive symptom severity as assessed by the Hamilton Rating Scale will serve as our primary study outcome. Secondary outcomes will include further indicators of mental health and changes in adaptive emotion regulation skills application. All outcomes will be assessed at intake and at 10 points in time over the course of the 15-month study period. Measures will include self-reports, observer ratings, momentary ecological assessments, and will be complemented in subsamples by experimental investigations and the analysis of hair steroids.

**Discussion:**

If findings should support the hypothesis that enhancing regulation skills reduces symptom severity in Major Depressive Disorder, systematic emotion regulation skills training can enhance the efficacy and efficiency of current treatments for this severe and highly prevalent disorder.

**Trial registration:**

This study is registered with ClinicalTrials.gov, number NCT01330485.

## Background

Major Depressive Disorder (MDD) is currently one of the most relevant mental health problems for individuals and societies. With life-time prevalence rates greater than 15% [1] and rates of chronicity around 20% [2], MDD ranks fourth among all medical and psychiatric disorders when considering disease burden, and worldwide it is the number one cause of disability [3]. Treatments for MDD have been shown to be effective [2,4,5]. However, previous outcome studies also indicate that many patients still suffer from residual symptoms [6] and are likely to relapse within two years after treatment [7]. These findings indicate the need to enhance current psychotherapeutic treatments for MDD.

In an attempt to improve upon existing treatments, research has recently focused on deficits in emotion regulation (ER) as risk factors for the development and maintenance of MDD [8-10]. ER refers to implicit or explicit processes involved in attempts to change the quality, the intensity, or the duration of undesired affective states in accordance with situational demands, biological needs, and individual goals [11-13]. Focusing on aspects relevant to clinical utilization, Berking and colleagues [8,14-16] have conceptualized adaptive ER as the situation-dependent interplay of the abilities to (1) become aware of, (2) identify and label, (3) gain proper understanding of, (4) adaptively modify or (5) accept and tolerate affective reactions, (6) approach and confront situations likely to trigger negative affects when necessary to attain personally relevant goals, and (7) provide compassionate self-support in distressing situations. These abilities have been proposed to help maintain a sense of control in distressing situations and thus reduce the risk of depression [4,14]. Adaptive ER skills are also believed to help prevent, reduce or shorten the intensity or duration of dysphoric states that have been found to reactivate depressive thinking patterns contributing to the (re-)occurrence of major depressive episodes [17,18].

Consistent with these assumptions, deficits in ER have been linked to depressive and other psychopathological symptoms; successful application of adaptive ER skills was positively related to indicators of mental health [19,20]. Longitudinal [21,22] and experimental [9,23,24] studies support deficits in ER as important antecedents of MDD. In treatment outcome studies, interventions that included work on ER deficits were shown to be effective in treatment for MDD, e.g., Dialectical Behavioral Therapy [25-27], Emotion-Focused Therapy [28,29], and Affect Regulation Training (ART) [8,14,15].

Among all treatments targeting ER, ART is likely the only transdiagnostic program that explicitly and exclusively aims to enhance general ER skills in at-risk and clinical populations. Previous studies have shown preliminary support for the efficacy of ART. In police officers, a group that had been shown to display significantly lower levels of adaptive ER competencies than the normal population, ART helped to enhance adaptive ER skills application [30]. In clinical settings, the ART program was evaluated in samples of inpatients. A randomly selected subgroup of patients meeting criteria for any mental disorder was offered to replace part of their regular cognitive behavioral therapy (CBT) with an abbreviated version of ART. Patients in the ART condition displayed significantly greater increases in adaptive ER, greater decreases in MDD and negative affect, and greater increases in positive affect than patients receiving only conventional CBT [19]. In a prospective randomized controlled trial on individuals meeting criteria for MDD [8], patients allocated to a condition in which some CBT sessions were replaced by a short version of ART also showed greater gains in the acquisition of health-relevant ER skills (modification, acceptance and tolerance of undesired emotions as well as effective self-support) and greater reductions of depressive symptoms when compared to patients in the regular CBT condition.

Despite these encouraging findings, existing research on ART is limited by a number of factors. First, previous clinical studies used a short version of ART. Second, ART has not yet been compared with an untreated control condition or with a condition that accounts for unspecific therapeutic factors. Third, it has not yet been investigated whether ART would augment the effects of other empirically-evidenced treatments for MDD (possibly because enhanced ER skills might allow patients to engage more intensely in the therapeutic process, [31]). Finally, in previous study outcomes the effectiveness of ART was exclusively assessed through self-report measures and only at pre- and post-treatment.

In an attempt to clarify whether experimentally enhancing general ER skills reduces depressive symptom severity and whether fostering adaptive ER skills enhances the outcome of subsequent individual CBT for depression (iCBT-D), we will evaluate the efficacy of ART in a prospective randomized controlled trial. Stand-alone and augmenting effects of ART will be compared with a waitlist control condition and a condition controlling for active ingredients common to most empirically evidenced treatments. Primary (depressive symptom severity) and secondary outcomes will be assessed at intake and 10 points over the course of the study. Measurements will include self-reports, observer ratings, ecological momentary assessments (EMA) [32] and will include experimental investigations and the analysis of hair steroids in subsamples.

## Methods/Design

**Figure 1:**
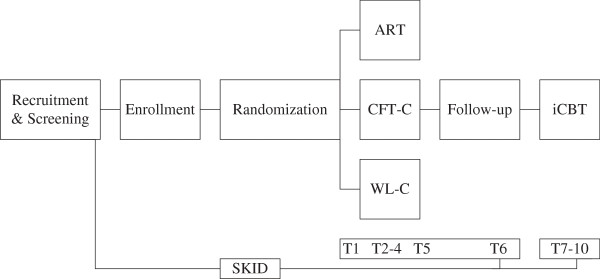
**Overview of design and assessments.** ART = Affect Regulation Training. CFT = Common Factor-based Treatment. WL = Wait-List. C = Control Group. iCBT = individual Cognitive Behavioral Therapy.

The study is designed as a prospective randomized controlled trial in an outpatient setting. Following three sessions with their individual therapists, enrolled patients will be assigned to the ART group, an active common factor-based treatment control condition (CFT-C) or a waitlist control group using a computerized randomization tool (randomisation.net). Group therapies will be provided for 18 hours over the course of 8 weeks. Participants in the waiting condition will be offered to participate in ART after completion of the study. Following the group and a 4-week follow-up waiting phase, all participants will receive 16 hours of standardized and manualized iCBT for depression. Individual treatment will be continued beyond the study if necessary. An overview of the study design is illustrated in Figure 1. The study has been approved by the ethical committee of the German Psychological Society and of the University of Marburg. It was registered with ClinicalTrials.gov, number NCT01330485.

### Inclusion and exclusion criteria

Inclusion criteria will include MDD as the primary diagnosis, age 18 or above, and sufficient German language skills. Exclusion criteria will include high risk of suicide, indication of substantial secondary gain (e.g., compensation issues), additional psychotherapeutic treatments, comorbid psychotic, substance-related, bipolar disorders, organic brain or other severe medical disorders, and severe cognitive impairments. Other comorbid disorders, including personality disorders, will be accepted to increase validity of the study.

### Recruitment

Study participants will be recruited at outpatient treatment centers in Marburg, Mainz, and Kassel. Potential participants will be screened on eligibility and provided with study information on the phone. Interested and potentially eligible patients will receive additional written information on the study and be invited to a Structured Clinical Interview for DSM-IV Axis I and II (SCID I, II) in one of the study centers. Patients meeting inclusion criteria and having provided informed consent will be included in the study. Recruitment will be conducted consecutively, that is, 10 to 15 individuals at a time will be enrolled in each cohort.

### Interventions

Interventions will include ART and CFT-C as group interventions and individual CBT for MDD.

### Affect Regulation Training (ART)

ART was developed as an adjunctive or stand-alone transdiagnostic and group-based intervention, explicitly focusing on an increase in adaptive ER in individuals who meet criteria for mental disorders or are at-risk for developing mental health problems. To foster effective ER, ART utilizes elements from various psychotherapeutic approaches as cognitive behavioral therapy [33], dialectical behavioral therapy [27] emotion focused therapy [29], mindfulness-based interventions [34], neuro-psychotherapeutic translational approaches [35], compassion-based therapy [36,37], problem solving therapies [38], and strength-focused interventions [39,40]. At the beginning of the training, participants are provided with information on emotions including biological and psychological origins, functions, risks, and benefits of emotional reactions. Then seven “vicious cycles” based on findings from the affective neurosciences and deemed important in the long-term maintenance of negative affect, are presented. Individuals are taught skills to break these cycles and enhance effective ER. These skills include muscle and breathing relaxation, nonjudgmental emotional awareness, acceptance, and tolerance, compassionate self-support, the identification of causes of emotional reactions, and modification of affective states. In the building of ER skills, special emphasis is placed on the importance of regular training. Additional information on ART is provided in the ART manual [14-16].

### Common Factor Treatment-Control (CFT-C)

CFT-C was established as an active control group to account for unspecific change mechanisms of psychotherapy (i.e., therapeutic alliance, resource activation, problem activation, motivational clarification, and problem solving) [35,39]. Following the identification of personally relevant goals and associated motives, acceptance is targeted for goals that cannot or no longer can be achieved; problem solving processes are initiated for achievable goals. Problem solving steps that are taught include the identification and a detailed description of problems and relevant situational features, the definition of goals, the development, evaluation, selection, and processing of solutions, and processes of success monitoring and reinitiating the problem solving or acceptance processes when necessary.

### Individual Cognitive Behavioral Therapy for Depression (iCBT-D)

Individual therapy will cover a 4-month period with 16 (weekly) 50-min sessions in total. Treatment will follow a manualized protocol based on procedures developed by Hautzinger [41], which includes psycho-education on MDD, behavioral activation, cognitive restructuring, social skills training, stress reduction, and relapse prevention.

### Assessments

Participants will be assessed at intake and at 10 points over the course of the study: before (T1), during (T2-4), and after the group-based phase (T5), after the subsequent 4-week follow-up waiting phase before individual CBT starts (T6), during (T7-9), and post the first four months of individual CBT (T10). Measures will include self-report questionnaires, interviews, observer-based ratings, EMA, experimental investigations, and analyses of hair steroids. Questionnaires will all be provided in paper pencil format and in German language. Interviews will be conducted face-to-face by intensively trained Master’s students majoring in clinical psychology who will closely be supervised by psychotherapists and psychotherapists in training for CBT, all of whom have Master’s degrees in clinical psychology. Participants will be provided with iPhones for the EMA. Over 7-day periods, time-contingent assessments will be taken 3 times per day and one hour after each of the three assessments. Participants will be given the chance to supplement time-contingent assessments with event-contingent assessments whenever they feel significantly distressed. Enrolled patients will also be asked to participate in an experimental investigation of ER skills and to provide hair probes. Participants will be compensated 50 Euros for the burden associated with study diagnostics, and an additional 20 Euros will be provided for participating in the experiment or providing hair probes. An overview of study variables, assessment points, and instruments is provided in Table 1.

**Table 1:** Overview of study variables and instruments

**Domain**	**Instrument**	**T0**	**T1**	**T2**	**T3**	**T4**	**T5**	**T6**	**T7**	**T8**	**T9**	**T10**
Depressive symptom severity (primary outcome)	Hamilton rating scale for depression		✕		✕			✕				✕
Diagnostics	Structured clinical interview for DSM-IV Axis I, II	✕						✕				✕
Stressors	List of situational stressors		✕	✕	✕	✕	✕	✕	✕	✕	✕	✕
Additional indicators of mental health	Beck depression inventory II		✕	✕	✕	✕	✕	✕	✕	✕	✕	✕
	Scales of psychological well-being		✕	✕	✕	✕	✕	✕	✕	✕	✕	✕
	Pos. and neg. affect schedule		✕	✕	✕	✕	✕	✕	✕	✕	✕	✕
	Short scales of affective states relevant for psychotherapy		✕	✕	✕	✕	✕	✕	✕	✕	✕	✕
	SHARP/others		✕				✕	✕				✕
	Brief symptom inventory		✕				✕	✕				✕
	Depression anxiety and stress scale		✕	✕	✕	✕	✕	✕	✕	✕	✕	✕
Emotion regulation	Emotion reg. skills questionnaire		✕	✕	✕	✕	✕	✕	✕	✕	✕	✕
	ERSQ- others		✕				✕	✕				✕
	Emotion reg. skills questionnaire- emotion specific		✕	✕	✕	✕	✕	✕	✕	✕	✕	✕
	ERSQ-ES- others		✕				✕	✕				✕
	Difficulties in emotion reg. scale		✕				✕	✕				✕
	Negative mood regulation scale		✕									
	Trait meta-mood scale		✕									
Confounding variables	Self-efficacy scale		✕				✕	✕				✕
	Multidim. perfectionism scale		✕				✕	✕				✕
	Rosenberg self-esteem scale		✕				✕	✕				✕
Additional assessments	Ecological momentary assessment		✕				✕					
	Laboratory experiment		✕				✕					
	Hair steroids		✕				✕					✕

### Primary outcome

The level of depressive symptom severity as assessed by the Hamilton Rating Scale for Depression (HRSD) [42] will serve as the primary study outcome. The HRSD is a clinician-administered semi-structured interview that assesses symptoms of MDD. Based on patients’ responses, clinicians rate the degree of 24 symptoms such as depressed mood, feelings of guilt, sleeping disturbances, and anxiety on 3-point or 5-point Likert scales. Higher sum scores indicate greater symptom severity. The cut-off points of 10, 19, 27, and 35 represent thresholds for mild, moderate, severe, and very severe depression, respectively. The HRSD is sensitive to change and corresponds well with overall clinical ratings of severity [43,44].

### Socio-demographic variables

The following socio-demographic data will be collected: age, gender, marital status, partnership, children, current living situation, educational level, occupation learned, occupation held, and immigration.

### Diagnostics

The Structured Clinical Interviews for DSM-IV Axis I and II (SCID I, II) [45] will be used to assess MDD and comorbid disorders. The SCID is a structured interview that assesses psychiatric diagnosis defined in the Diagnostic and Statistical Manual of the American Psychiatric Association, 4th edition (DSM-IV). The SCID II includes a screening questionnaire to limit the number of questions of the subsequent interview. DSM-5 diagnosis for MDD will be added when possible.

### Stressors

The List of Situational Stressors (LSS) will assess for potential cues of negative emotional reactions to 11 daily events. Individuals are asked to rate how often within the last week they experienced stressors such as arguments with a friend, romantic partner or family member (interpersonal domain), high workload (work-related), financial problems (financial domain), and trouble with means of transport (everyday stressors). The scale has previously been used in a study on affective reactivity as a predictor of depressive symptoms [46].

### Additional indicators of mental health

The Beck Depression Inventory II (BDI II) [47] will be included in this study as a secondary measure of depressive symptom severity. The BDI II is a widely used 21-item self-report measure of somatic, behavioral, emotional, and cognitive signs of depression. Good reliability and validity have previously been demonstrated [48].

An unpublished German translation of the Scales of Psychological Well-Being (SPWB) [49] will be used as an indicator of psychological well-being. The SPWB includes scales for autonomy, environmental mastery, personal growth, positive relations with others, purpose in life, and self-acceptance. Given that psychometric analyses of the SPWB have not always supported the proposed six factor structure [50,51], we will use the total score in this study. High internal consistency and test-retest reliability coefficients were reported in the original validation study [49].

Positive and negative affective states will be assessed using the Positive and Negative Affect Schedule (PANAS) [52]. On 5-point Likert scales (0 = not at all to 4 = almost always), participants are asked to rate the frequency of 20 affective states. Within a previous validation study [52], good internal consistency was revealed, and associations with related constructs such as anxiety, depression, and neuroticism were significant and in the expected directions. To assess more specific emotional reactions, Berking developed the Short Scales for the Assessment of Affective States Relevant in Psychotherapeutic Treatments- Self/Other (SHARP). On 4-point Likert scales, participants rate how often they experienced 50 different emotions in the past week.

To obtain continuous information on comorbid symptom severity, a global severity index will be computed on all but the depression scales of the Brief Symptom Inventory (BSI) [53]. The BSI is a screening tool for psychological disturbance including depression, somatization, obsessive-compulsive symptoms, interpersonal sensitivity, anxiety, hostility, phobic anxiety, paranoid ideation, and psychoticism. Adequate psychometric properties with very high internal consistency for the total score have previously been reported for the German scale [54]. Accounting for high comorbidity between MDD and anxiety disorders [55], the Depression Anxiety and Stress Scale (DASS-21) [56] will also be administered. The DASS is a 42-item self-report instrument designed to measure the three related negative emotional states of depression, anxiety and stress. To our knowledge, no validation study of the German DASS has been published to date. For the English scale, good to excellent internal consistency scores were reported, and associations with other measures of depression, anxiety, and stress were within the high range [57].

### Emotion regulation

Successful application of adaptive ER skills will be assessed through self-reports and observer-based ratings using the Emotion Regulation Skills Questionnaire-Self/Other (ERSQ) [20]. The ERSQ is a 27-item measure addressing the application of nine competencies included in the ART model of effective ER (i.e., Awareness, Modification, Acceptance, Understanding, Sensations, Clarity, Self-Support, Readiness to Confront, and Tolerance). Results from validation studies [20] indicate that both the total score and the subscales of the ERSQ have good internal consistencies and adequate retest-reliability. Associations with other scales supported the scale’s convergent and discriminant validity [20]. To assess ER with regard to specific affective states, the Emotion Regulation Skills Questionnaire- Emotion Specific-Self/Other (ERSQ-ES) [58] will be used. Sound psychometric properties have previously been reported for this scale. The observer-based versions of the ERSQ and ERSQ-ES were developed for the purpose of this study and will be evaluated on the basis of study data.

Further applied ER scales with established psychometric properties include a German translation of the Difficulties in Emotion Regulation Scale (DERS) [59], the Negative Mood Regulation Scale (NMR) [60], and the Trait Meta-Mood Scale (TMMS) [61]. The DERS assesses difficulties in emotional awareness, emotional acceptance, goal-directed behaviors and the application of effective ER strategies. The NMR was designed as a measure of individuals’ general expectancies that emotional states can effectively be changed through ways of behaving or thinking. The TMMS is a measure of emotional intelligence including the components of emotional attention, clarity, and mood repair.

### Confounding variables

To control for potential confounds, we will assess general self-efficacy with a 10-item, validated German scale (Skala zur Allgemeinen Selbstwirksamkeitserwartung, ASE) [62]. Perfectionism will be assessed by the Multidimensional Perfectionism Scale (MPS) [63], including 35 items on concerns about mistakes, personal standards, parental expectations, parental criticism, doubts about actions, and organization. The Rosenberg Self-Esteem scale (RSE) [64] will be used as a measure of self-esteem. Adequate psychometric properties of these scales have been reported in the cited studies.

### Other outcome measures

Targeting the issue of cognitive distortion and to increase ecological validity, EMA will be implemented as a real-time assessment of ER, affective states, and affective changes. Within EMA, affective states and ER will be assessed by short scales of the SHARP and ERSQ-ES. Additional questions will address location, activity, and interaction partners. To reduce self-report biases and to yield further information on causality, the effects of adaptive ER on positive and negative affect will be tested experimentally. In laboratory settings, negative and positive affective states will be induced by music and self-related statements using the Velten [65] method. Participants will be provided with oral instructions of adaptive ER strategies (i.e., acceptance, positive reappraisal, compassionate self-support, positive appreciation). Hair probes will be taken to gather data for biological features of MDD. Elevated levels of hair steroids (i.e., cortisol and cortisone) have frequently been linked to MDD [66]. In the analyses of hair steroids, we will use liquid chromatography with linked tandem mass spectrometry.

### Participants

Sample size for the group phase was set to *N* = 120 (*n* = 40 per condition). A minimum of *n* = 90 individuals (*n* = 30 per condition) is expected to complete individual CBT. Targeted sample sizes are based on power calculations. The intended sample of 120 individuals can be expected to provide sufficient power to detect small effects [67].

### Statistical analyses

Data will be analyzed according to intent to treat and treatment completers principles. The intent to treatment analyses will be the primary level of analyses. Mixed effect modeling will serve as the main approach in the analyses of study data and in the treatment of missing values.

Growth curves of ER and depressive symptom severity will be computed per group on the basis of assessments before (T1-T5) and during individual CBT (T6-T10) to test for stand-alone and augmentation effects of ART. Slopes for the ART condition are hypothesized to be larger than those for the active or waitlist control groups, indicating the effectiveness of adaptive ER skills application enhancement and a decrease in depressive symptom severity by the ART program. In the secondary analyses, we will test for ART effects on well-being, positive, and negative affect. Bootstrapping enhanced, multilevel mediation analyses [68] will be conducted to test ER as a mediator of health improvements in the ART condition. Analyses will be conducted computing a total score of adaptive ER and separately for the different ART skills to understand the importance of specific ER skills. Moderated mediation analyses [69] will be used to examine the importance of specific ER skills in the mediation of positive health outcomes. Latent growth curve and latent change score modeling [70] will further be used in the investigation of reciprocal associations between levels and changes of ER and health outcomes. Comorbid symptom severity and potential confounding variables (i.e., general self-efficacy, perfectionism, and self-esteem) will be included in the analyses as moderating variables. The analyses of EMA and the experimental and biological data will serve to increase the validity of the ART evaluation. SEM-based Multi-Trait, Multi-Method approaches will be applied in the investigation of psychometric properties and in the comparison of outcomes by different assessment methods.

## Discussion

We should find support for our hypothesis that systematically enhancing general ER skills helps reduce depressive symptoms in individuals meeting criteria for MDD, given that deficits in ER are commonly considered a relevant risk and maintaining factor for this disorder. Former studies on ART, a program explicitly and exclusively focusing on an increase in adaptive ER skills application, have provided preliminary support for this assumption [8,19]. However, the validity of these studies has been compromised through a number of limitations such as the lack of an untreated control condition or a condition that controls for unspecific therapeutic factors, the lack of testing for augmentation effects of the ART on other treatments such as individual CBT for depression, and the exclusive use of self-reports at pre- and post-treatment. To improve on these limitations of previous studies and to advance the literature on associations between ER and MDD, we will systematically evaluate ART in a prospective randomized controlled trial.

This study will provide significant contributions to the literature. First, by controlling for time and unspecific treatment effects, the results will provide insight into the effects of enhancing the application of adaptive ER skills on symptoms of MDD. Second, from the two-phase design of this study that includes a period of individual CBT following the group phase, the stand-alone effects of ART on ER and depressive symptom severity can be tested, as well as the augmentation effects of ART on CBT outcomes. Third, the application of various measures, including self-reports, observer-based ratings, momentary assessments, experimental investigations, and the analysis of hair probes, will augment the reliability of findings and help to overcome methodological restrictions such as cognitive distortion and social desirability in retrospective testing and self-reports. The implementation of various assessment points over the course of the study will allow for detailed analyses of changes in and associations between ER and depressive symptom severity.

In light of the methodological strengths, the present study has the potential to substantially increase knowledge on ER processes that underlie and maintain MDD. Given the likely importance of deficits in ER across disorders, this study might also yield a better understanding of comorbidity in MDD, which currently is one of the major challenges in the treatment of depressive disorders. Therapeutically, fostering adaptive ER skills application by ART might substantially improve treatment outcomes for individuals with depressive and possibly also comorbid disorders.

## Competing interests

The authors declare that they have no competing interests.

## Authors’ contributions

All authors have made substantial contributions to the conception and execution of the study. The Affect Regulation Training was developed by MB. MB, WH and WR were responsible for acquiring funding for the study. AME, JK, WR, and MB will be conducting the study in Marburg, WH and MB will supervise the study in Mainz, and the study will be supervised by MB in Kassel. AME and MB drafted the manuscript. All authors revised it critically and have given final approval for the version to be published.

## Pre-publication history

The pre-publication history for this paper can be accessed here:

http://www.biomedcentral.com/1471-244X/14/20/prepub
